# Synergistic Cytotoxicity of Methyl 4-Hydroxycinnamate and Carnosic Acid to Acute Myeloid Leukemia Cells *via* Calcium-Dependent Apoptosis Induction

**DOI:** 10.3389/fphar.2019.00507

**Published:** 2019-05-09

**Authors:** Aviram Trachtenberg, Suchismita Muduli, Katarzyna Sidoryk, Marcin Cybulski, Michael Danilenko

**Affiliations:** ^1^Department of Clinical Biochemistry and Pharmacology, Ben-Gurion University of the Negev, Beer Sheva, Israel; ^2^Chemistry Department, Pharmaceutical Research Institute, Warsaw, Poland

**Keywords:** acute myeloid leukemia, curcumin, carnosic acid, methyl 4-hydroxycinnamate, calcium-dependent apoptosis

## Abstract

Acute myeloid leukemia (AML) is a malignant hematopoietic disease with poor prognosis for most patients. Conventional chemotherapy has been the standard treatment approach for AML in the past 40 years with limited success. Although, several targeted drugs were recently approved, their long-term impact on survival of patients with AML is yet to be determined. Thus, it is still necessary to develop alternative therapeutic approaches for this disease. We have previously shown a marked synergistic anti-leukemic effect of two polyphenols, curcumin (CUR) and carnosic acid (CA), on AML cells *in-vitro* and *in-vivo*. In this study, we identified another phenolic compound, methyl 4-hydroxycinnamate (MHC), which among several tested phytochemicals could uniquely cooperate with CA in killing AML cells, but not normal peripheral blood mononuclear cells. Notably, our data revealed striking phenotypical and mechanistic similarities in the apoptotic effects of MHC+CA and CUR+CA on AML cells. Yet, we show that MHC is a non-fluorescent molecule, which is an important technical advantage over CUR that can interfere in various fluorescence-based assays. Collectively, we demonstrated for the first time the antileukemic activity of MHC in combination with another phenolic compound. This type of synergistically acting combinations may represent prototypes for novel antileukemic therapy.

## Introduction

Acute myeloid leukemia (AML) is a devastating hematological malignancy characterized by poor survival, particularly for older patients, and high relapse rate. In the past four decades, there have been no major changes in the standard AML chemotherapy regimen. Although in 2017–2018 eight new promising AML drugs were approved by the U.S. Food and Drug Administration (FDA) (Stone, 2017; Rowe, 2018), their impact on long-term patient survival is yet to be determined. Various natural and synthetic phenolic compounds have been shown to possess anti-leukemic potential in preclinical models (De Martino et al., 2011), curcumin (CUR) being one of the most studied polyphenols (Kelkel et al., 2010). However, the majority of *in vitro* studies employed high supraphysiological concentrations of CUR (Lin et al., 2008; Zhang et al., 2017; Martínez-Castillo et al., 2018) which induced generalized cellular stress events leading to cell death, such as oxidative stress (Lin et al., 2008; Yoon et al., 2012), ER stress (Lin et al., 2008; Cao et al., 2013), or mitochondrial damage (Yoon et al., 2012; Cao et al., 2013; Xu et al., 2015). We have previously shown that CUR combined with another polyphenol, carnosic acid (CA), at noncytotoxic concentrations of each agent synergistically and selectively killed AML cells by inducing massive apoptosis both *in vitro* and *in vivo* (Pesakhov et al., 2010, 2016). This synergistic effect was not accompanied by cellular stress and was specifically mediated by cytosolic calcium ([Ca^2+^]_cyt_) overload (Pesakhov et al., 2010, 2016).

In the current study, we examined whether in addition to CUR some other phytochemicals are capable of synergizing with CA against AML cells. Thus, following screening of several phenolic compounds and a sesquiterpene lactone we identified another synergistically acting combination comprised of the phenolic acid derivative methyl 4-hydroxycinnamate (MHC) and CA. MHC is found in several plants, such as green onion (*Allium cepa*) (Xiao and Parkin, 2007) or noni (*Morinda citrifolia* L.) leaves (Zhang et al., 2016), and has potential chemopreventive activity (Xiao and Parkin, 2007). To the best of our knowledge, MHC has not been previously tested as an antileukemic agent. Here, we report that the cytotoxic effect of the MHC+CA combination is very similar to that of CUR+CA, both phenomenologically and mechanistically. Furthermore, in contrast to CUR, which is highly fluorescent, and thus is known to interfere with fluorescence-based assays (Nelson et al., 2017), MHC had no detectable fluorescence when tested by flow cytometry in a wide range of wavelengths (FL1–FL10).

## Materials and Methods

### Materials

Curcumin (≥90%) and carnosic acid (98%) were purchased from Cayman Chemicals (Ann Arbor, MI, USA) and Chemlin UK (Nanjing, China), respectively. Methyl 4-hydroxycinnamate (96%) was synthesized by Dr. Katarzyna Sidoryk (Chemistry Department, Pharmaceutical Research Institute, Poland), as described previously (Sidoryk et al., 2018). zVAD-fmk was purchased from AdooQ BioScience (Irvine, CA, USA). Propidium iodide (PI), 2-aminoethoxydiphenyl borate (2-APB), Arabinosylcytosine (Ara-C), and staurosporine (STS) were purchased from Sigma (Rehovot, Israel). Annexin V-APC was obtained from BioLegend (San Diego, CA, USA). Fluo-3/AM, 2′,7′-dichlorofluorescein-diacetate (DCFH-DA), dihydrorhodamine 123 (DHR) and tetramethylrhodamine methyl ester (TMRE) were purchased from Santa Cruz Biotechnology (Dallas, TX, USA). RPMI 1640 medium and heat-inactivated fetal bovine serum (FBS) were purchased from Gibco-Invitrogen (Carlsbad, CA, USA). Hank's buffered salt solution (HBSS), Ca^2+^/Mg^2+^-free phosphate buffered saline (PBS), penicillin, streptomycin, and HEPES were purchased from Biological Industries (Beit Haemek, Israel). Stock solutions of curcumin (5 mM) and carnosic acid (10 mM) were prepared in absolute ethanol and methyl 4-hydroxycinnamate (50 mM) in DMSO.

### Cell Culture and Enumeration

Human AML cell lines, such as KG-1a stem-like cells (CCL-246.1) and HL60 myoblastic cells (CCL-240), were purchased from American Type Culture Collection (Rockville, MD). Cells were cultured in RPMI 1640 medium supplemented with 10% FBS, penicillin (100 U/ml), streptomycin (0.1 mg/ml), and 10 mM HEPES (pH = 7.4) in a humidified atmosphere of 95% air and 5% CO_2_, at 37°C. Cells were enumerated in Vi-Cell XR cell viability analyzer (Beckman Coulter Inc., Fullerton, CA) using an automatic trypan blue exclusion assay. The number of viable (trypan blue-impermeable) cells was counted directly, and cell viability was calculated as the percentage of viable cells relative to the total (viable + dead) cell count.

### Acridine Orange and Ethidium Bromide Staining

Cells were collected by centrifugation and double stained with 14 μg/ml acridine orange and 14 μg/ml ethidium bromide, as described previously (Pesakhov et al., 2010). Nuclear morphology of stained cells was examined by fluorescent microscopy at a magnification of 400x.

### Annexin V/Propidium Iodide Assay

Cells were washed with PBS then stained with annexin V-APC and PI, as described previously (Pesakhov et al., 2016). Percentages of apoptotic cells were determined by flow cytometry in a Gallios instrument (Beckman Coulter, Miami, FL). For each analysis 10,000 events were recorded, and the data were processed using Kaluza software, version 2.1 (Beckman Coulter).

### Determination of Intracellular Levels of Reactive Oxygen Species (ROS)

The intracellular ROS levels were determined as described previously (Pesakhov et al., 2010) using the oxidation-sensitive fluorescent probes DCFH-DA and DHR. Cells were harvested, washed with HEPES-supplemented HBSS (pH = 7.3) and loaded with 5 μM DCFH-DA or DHR. Cells were then incubated in a shaking water bath at 37°C, for 15 min in the dark. Fluorescence intensity was analyzed by flow cytometry, as described above.

### Determination of Mitochondrial Membrane Potential

Cells were harvested, washed with HEPES-supplemented HBSS (pH = 7.3) and loaded with TMRE (100 nM), for 30 min in the dark, washed and resuspended in serum-free medium. Changes in mitochondrial membrane potential were assessed by flow cytometry.

### Measurement of Cytosolic Calcium Levels ([Ca^2+^]_cyt_)

To evaluate changes in steady-state [Ca^2+^]_cyt_, cells were harvested, washed and incubated with 2.5 μM Fluo-3/AM in calcium (2 mM)-supplemented Ringer's solution (Levin-Gromiko et al., 2014; Pesakhov et al., 2016) at room temperature for 30 min in the dark. Cells were then washed, resuspended in Ca^2+^-free Ringer's solution and analyzed by flow cytometry.

### Western Blot Analysis

Western blotting was performed using whole cell extracts, as described before (Pesakhov et al., 2016). The following primary antibodies were used: caspase-3 from Santa Cruz Biotechnology (sc-7272; 1:500); cleaved caspase-3 from Cell Signaling Technology (#9661; 1:1,000) and poly(ADP-ribose) polymerase (PARP) from Enzo (BML-SA253; 1:5,000).

### Statistical Analysis

All experiments were conducted at least three times. Statistically significant differences between two experimental groups were estimated by unpaired two-tailed Student's *t*-test. The significance of the differences between the means of several subgroups was assessed by one-way ANOVA with Tukey multiple comparison *post-hoc* analysis. A *P* < 0.05 was considered statistically significant. The synergy between the effects of two compounds was determined as described previously (Danilenko et al., 2001; Pesakhov et al., 2010). Briefly, two compounds (A and B) were considered to show enhancement in the particular experiment if the effect of their combination (AB) was larger than the sum of their individual effects (AB>A+B), the data being compared after subtraction of the respective control values from A, B, and AB. Detailed analysis of the interaction between two compounds was performed by the combination index (CI) method (see Supplementary Material). The statistical analyses were performed using GraphPad Prism 6.0 software (Graph-Pad Software, San Diego, CA).

## Results

### MHC and CUR, but Not Other Tested Phytochemicals, Similarly Synergize With CA to Induce Apoptotic Cell Death in AML Cells

**Figure 1:**
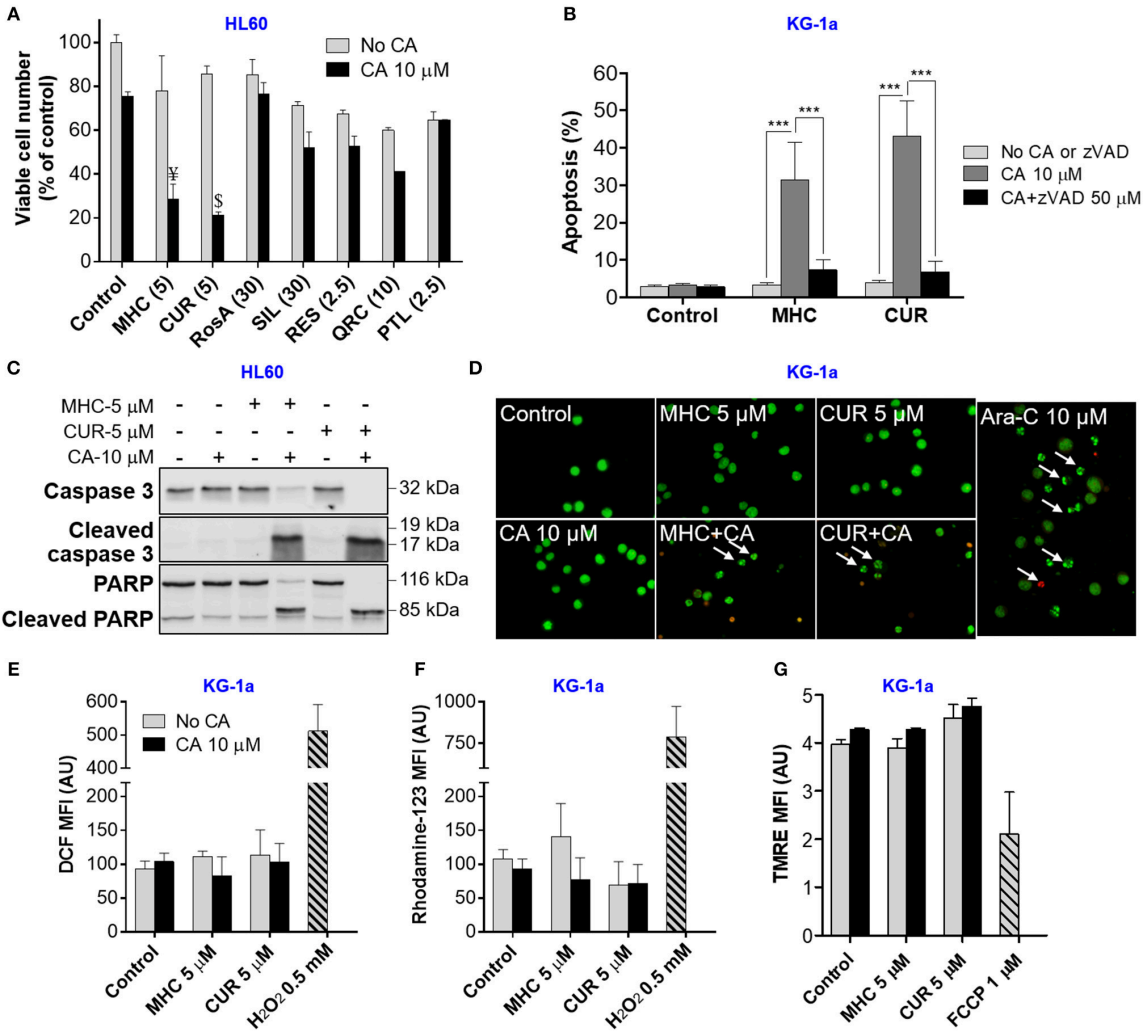
MHC is similar to CUR in the ability to synergize with CA in inducting anti-leukemic effects on AML cells. **(A)** Changes in viable cell numbers. HL60 cells were treated with different phenolic compounds (in μM): CUR, MHC, rosmarinic acid (RosA), silibinin (SIL), resveratrol (RES), quercetin (QRC), parthenolide (PTL) and/or 10 μM CA, for 72 h. Viable cell numbers were determined by the trypan blue exclusion assay. The data are the means ± SD (*n* = 3). **(B)** Apoptosis induction. KG1a cells were treated for 8 h in the presence or absence of zVAD (50 μM). The extent of apoptosis was determined by the annexin V/PI binding assay. **(C)** Western blot analysis of caspase-3 and PARP cleavage in HL60 cells treated with MHC or CUR with or without CA, for 24 h. **(D)** Changes in nuclear morphology. KG-1a cells were treated with the indicated compounds, for 8 h, followed by staining with acridine orange (green fluorescence) and ethidium bromide (red fluorescence). Stained cells were examined under fluorescence microscope at 400x magnification. Cells treated with 10 μM Ara-C, for 8 h, were used as the positive control. Arrows indicate late apoptotic cells and/or apoptotic bodies. **(E,F)** Assessment of the intracellular ROS. KG-1a cells were treated with the indicated compounds, for 4 h, followed by loading with DCFH_2_-DA **(E)** or DHR **(F)**. DCF and rhodamine-123 fluorescence was measured by flow cytometry. Averaged geometric means of fluorescence intensities (MFI) ± SD from three independent experiments are shown. H_2_O_2_ (0.5 mM) was used as the positive control. **(G)** Assessment of the mitochondrial membrane potential. KG-1a cells were treated with the indicated compounds, for 2 h, followed by loading with TMRE. Averaged MFI ± SD were measured in 3 independent experiments. The uncoupler FCCP (1 μM, 10 min) was used as the positive control. Synergistic effect (AB>A+B): ¥, *p* < 0.05; $, *p* < 0.01; Student's *t*-test. AU, arbitrary units. **(B)** ***p < 0.001; one-way ANOVA with Tukey multiple comparison *post-hoc* analysis.

We first performed pilot experiments in HL60 cells to determine the maximal non-cytotoxic concentrations (MNC) of several phytochemicals (Supplemental Figure S1) for further screening of these compounds for the ability to cooperate with CA in inducing AML cell death. Figure 1A demonstrates that, when applied alone at the MNC for 72 h, the phenolic compounds MHC, CA, rosmarinic acid (RosA), silibinin (SIL), resveratrol (RES), quercetin (QRC), and the sesquiterpene lactone parthenolide (PTL) reduced HL60 viable cell numbers to a varying extent (by 10–40%), without significantly affecting cell viability (Supplemental Figure S1). However, besides CUR, only MHC could strongly synergize with CA in reducing viable cell numbers, as determined in KG-1a, HL60, and U937 AML cells (Figure 1A and Supplemental Figures S2A,D). The analysis of interaction between MHC and CA in KG-1a cells shows a strong synergy at different concentrations of the two compounds, as indicated by very low (< < 1) Combination Index values (Supplemental Figures S2B,C). A synergistic decrease in cell viability, i.e., in the percentage of viable cells relative to the total cell count, was observed only in samples treated with MHC+CA or CUR+CA (Supplemental Figure S1), though this effect was less pronounced compared to a decrease in viable cell numbers (Figure 1A). This may be due to inhibition of cell proliferation in addition to cytotoxicity. Furthermore, some of the cells can be completely destroyed following combined treatments, a phenomenon not uncommon in cell culture studies. Thus, the proportion of dead cells in cultures remaining after CUR+CA and MHC+CA treatments (Figure 1A) might be underestimated when using trypan blue exclusion assay (Supplemental Figure S1).

In analogy with the previously characterized CUR+CA combination (Pesakhov et al., 2010, 2016), MHC+CA-induced cytotoxicity was accompanied by an induction of caspase-dependent apoptosis (Figure 1B and Supplemental Figure S3B) that was manifested by a rapid zVAD-inhibitable cleavage of caspase 3 and PARP (Figure 1C and Supplemental Figures S2E, S3A). Notably, although the combinations were cytotoxic to all three cell lines tested (Figure 1A and Supplemental Figures S2A,D), KG-1a cells were the most sensitive, as demonstrated by a more rapid cleavage of caspase-3 or PARP **(**8 h; Supplemental Figure S3A) than in HL60 and U937 cells (24 h; Figure 1C and Supplemental Figure S2E). In contrast, neither combination significantly affected cell death in PBMC samples. This was evidenced both by the annexin V/PI assay (Supplemental Figure S4A) and by continuous real-time monitoring for the appearance of low-area (shrunk) cells in the IncuCyte Live-Cell Analysis System (Supplemental Figures S2F, S4B,C). U937 cells treated with MHC+CA and PBMCs treated with staurosporine, a known apoptosis inducer, exhibited a marked time-dependent increase in the proportion of low-area cells (Supplemental Figures S2F, S4B,C and Supplemental Videos) which are considered apoptotic/dead (Majno and Joris, 1995).

As expected, apoptosis of AML cells was accompanied by shrinkage and fragmentation of the nuclei and chromatin condensation (Figure 1D). On the other hand, we have not observed the signs of necrotic cell death, such as uniformly stained (in orange) nuclei of regular size or larger, which usually exhibit normal morphology. Importantly, the induction of apoptosis was not preceded by intracellular ROS accumulation (Figures 1E,F) or changes in the mitochondrial membrane potential (Figure 1G).

### A Synergistic Induction of Apoptosis by the MHC+CA Combination Is Mediated by Intracellular Calcium Accumulation

**Figure 2:**
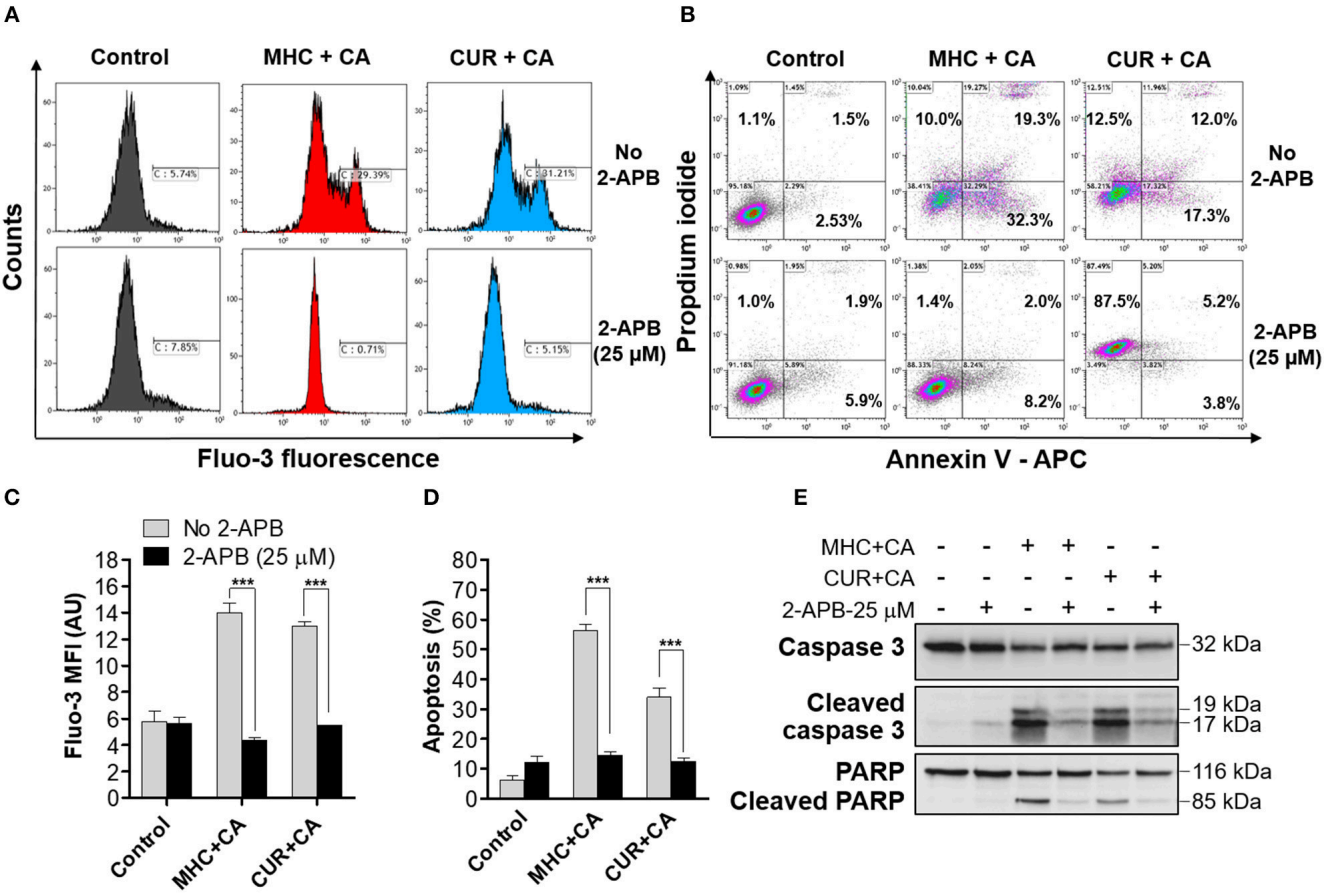
Similar to CUR+CA, the MHC+CA combination induces calcium-dependent apoptosis in the KG-1a cell line. Cells were treated with MHC+CA or CUR+CA in the presence or absence of 2-APB (25 μM). **(A,C)** Steady-state cytosolic calcium levels were measured after 4 h of incubation. **(A)** Typical histograms obtained from Fluo-3 loaded cells in a representative experiment. **(C)** Averaged Fluo-3 MFI ± SD from three independent experiments. **(B,D,E)** The extent of apoptosis was determined by the annexin V/PI binding assay, after 8 h of incubation. **(B)** Typical flow cytometric data obtained in a representative experiment. The data were analyzed after setting threshold for CUR+CA auto-fluorescence. **(D)** Averaged percentages of apoptotic cells ± SD (*n* = 3). **(E)** Representative Western blots showing caspase-3 and PARP cleavage (*n* = 3). ***p < 0.001; one-way ANOVA with Tukey multiple comparison *post-hoc* analysis.

Since CUR+CA-induced apoptosis was previously found to be mediated by a sustained [Ca^2+^]_cyt_ overload, we next examined whether MHC+CA also kills AML cells by a Ca^2+^-dependent mechanism (Pesakhov et al., 2016). Indeed, using the cytosolic Ca^2+^ indicator Fluo-3 we found that, similar to CUR+CA, the MHC+CA combination applied to KG-1a cells caused a sustained elevation of [Ca^2+^]_cyt_ and that this effect was prevented by 2-APB known to antagonize inositol trisphosphate receptors (IP_3_R) and store-operated Ca^2+^ channels (Figures 2A,C) (Dobrydneva and Blackmore, 2001; Yanamandra et al., 2011; Littlechild et al., 2015). Likewise, 2-APB abrogated the induction of apoptosis in MHC+CA-treated cells (Figures 2B,D). The latter inhibitory effect was associated with an almost complete block of MHC+CA-induced caspase-3 and PARP cleavage by 2-APB (Figure 2E).

### Fluorescence Properties of CUR and MHC

CUR is known as a fluorescent molecule and is considered a pan-assay interference compound (PAINS) (Nelson et al., 2017). Furthermore, CUR is unstable in aqueous solutions (Wang et al., 1997) and various antioxidants, including phenolic compounds, have been shown to stabilize CUR in buffers and to increase its plasma levels in animals (Nimiya et al., 2016; Nelson et al., 2017). Screening of CUR-treated and otherwise unstained AML cell samples over the entire standard set of flow cytometer channels (FL1–FL10) revealed noticeable CUR fluorescence in half of these channels, FL1–FL3 and FL9–FL10 (Supplemental Figures S5A,B). Furthermore, CUR fluorescence was found to increase in the presence of the antioxidant CA (Supplemental Figure S5C). This made it necessary to utilize fluorophores, which can be detected in the “CUR-insensitive” range of wavelengths, or, if not possible, to set threshold fluorescence parameters using unstained CUR ± CA-treated cells. For instance, in the annexin V/PI assay we utilized allophycocyanin (APC)-labeled annexin V (e.g., Figure 2B and Supplemental Figure S3B) for measurements in FL6, instead of the most commonly used fluorescein isothiocyanate (FITC)-labeled annexin V which has green fluorescence detected in FL1. Threshold for orange CUR+CA fluorescence in the propidium iodide (FL3) channel was set as described above (Figure 2B and Supplemental Figure S3B). Interestingly, we found that 2-APB strongly enhanced CUR+CA fluorescence in PI-stained cells (Figure 2B). In contrast to CUR, MHC did not exhibit auto-fluorescence under any experimental conditions of this study (Figure 2B, Supplemental Figures S3B, S5), and thus did not require any adjustments in fluorescence-based assays.

## Discussion

The major novel finding of this study is that, similar to CUR (Pesakhov et al., 2016), MHC is capable of synergistically cooperating with another phenolic compound, CA, at low concentrations of each agent in inducing profound cytotoxicity to AML cells, but not to normal PBMCs (Supplemental Figure S4) *in vitro*. This similarity was evidenced by a comparable ability of the two combinations to induce caspase- and calcium-dependent apoptosis (Figures 1B, 2B,D and Supplemental Figures S2E, S3) which was not associated with increased generation of ROS (Figures 1E,F) or reduction in the mitochondrial membrane potential (Figure 1G). Interestingly, no such cooperation was observed between MHC and CUR (data not shown) or between CA and other phytochemicals tested here (Figure 1A and Supplemental Figure S1). These findings suggest that comparable apoptosis-inducing activities of MHC+CA and CUR+CA may be due to certain structural similarities between MHC and CUR molecules, such as the presence of the 4-hydroxyl group at the aromatic ring and the α,β-unsaturated carbonyl group (Supplemental Figure S6). Apart from the above similarities, we found that MHC is a non-fluorescent molecule (Supplemental Figure S5) which made it technically superior to CUR, a widely recognized PAINS compound (Nelson et al., 2017). Further mechanistic and translational studies are needed to evaluate the therapeutic potential of the MHC+CA combination. This and similar combinations of synergistically acting natural or synthetic phenolic compounds may represent molecular prototypes for the design of novel modalities in AML therapy and/or prevention.

## Data Availability

All datasets generated for this study are included in the manuscript and/or the Supplementary Files.

## Author Contributions

MD, AT, and KS conceived the concept. AT and MD designed experiments and wrote the manuscript. AT and SM performed experiments. KS and MC provided reagents.

### Conflict of Interest Statement

The authors declare that the research was conducted in the absence of any commercial or financial relationships that could be construed as a potential conflict of interest.

## Abbreviations

2-APB: 2-aminoethoxydiphenyl borate
Ara-C: arabinosylcytosine (Cytarabine)
DCF, 2′: 7′-dichlorofluorescein
DCFH-DA, 2′: 7′-dichlorofluorescein-diacetate
DHR: dihydrorhodamine 123
FCCP: carbonyl cyanide-4-(trifluoromethoxy)phenylhydrazone
IP_3_R: inositol trisphosphate receptor
PARP: poly(ADP-ribose) polymerase
PBMC: peripheral blood mononuclear cells
PI: propidium iodide
STS: staurosporine
TMRE: tetramethylrhodamine methyl ester.
