# Supplementary material for: Synergistic Cytotoxicity of Methyl 4-Hydroxycinnamate and Carnosic Acid to Acute Myeloid Leukemia Cells via Calcium-Dependent Apoptosis Induction
Source: Front Pharmacol. 2019 May 9;10:507. doi: 10.3389/fphar.2019.00507 (PMC6521573; doi:10.3389/fphar.2019.00507)
Supplement: Supplementary file 1 [file Table_1.docx]

Supplementary Material

Synergistic cytotoxicity of methyl 4-hydroxycinnamate and carnosic acid to acute myeloid leukemia cells *via* calcium-dependent apoptosis induction

Aviram Trachtenberg^1*^, Suchismita Muduli^1^, Katarzyna Sidoryk^2^, Marcin Cybulski^2^, Michael Danilenko^1^

^1^Ben-Gurion University of the Negev, Department of Clinical Biochemistry and Pharmacology, Beer Sheva 84105, Israel

^2^Chemistry Department, Pharmaceutical Research Institute, Warsaw 01-793, Poland

*** Correspondence:** Aviram Trachtenberg, Department of Clinical Biochemistry and Pharmacology, Faculty of Health Sciences, Ben-Gurion University of the Negev, P.O. Box 653, 84105 Beer Sheva, Israel. E-mail: aviramtr@post.bgu.ac.il

## Supplemental Materials and Methods

**Cell culture**

Human U937 myelomonocytic leukemia cells (CRL-1593.2) were purchased from American Type Culture Collection (Rockville, MD). Samples of peripheral blood were collected with informed consent from healthy adult donors upon the approval by the institutional Helsinki committee (Soroka University Medical Center, Beer Sheva, Israel). Peripheral blood mononuclear blood cells (PBMCs) were isolated as described previously ([Pesakhov et al., 2016](#_ENREF_1)) using Histopaque-1077 gradient centrifugation.

**Combination index analysis**

## The interaction between MHC and CA in inducing cytotoxicity was assessed by the combination index (CI) analysis using CompuSyn 1.0 software (ComboSyn, Inc.). The CI values were calculated based on the extent of reduction in viable cell numbers (fraction affected) by each agent individually and by the combination at non-constant ratios. CI values of <1, 1, and >1 represent synergism, additivity and antagonism, respectively.

**Assessment of U937 and PBMC cell viability by the IncuCyte cell-by-cell analysis**

Cell viability was assessed by counting cells with a normal cellular area (≥ 100 μm^2^ for U937 cells; ≥ 50 μm^2^ for PBMC) and low cellular area (< 100 μm^2^ for U937 cells; < 50 μm^2^ for PBMC) using the IncuCyte® S3 Live-Cell Analysis System (Essen BioScience). The low-area cells may appear as shrunk dead cells or apoptotic bodies.

Cells were seeded in a 24-well plate and incubated 14-24 h under standard cell culture conditions. Nine fields of view (at 200x magnification) per well were automatically photographed at different time points, and the percentage of low-area cells in each field was calculated by the cell-by-cell analysis using IncuCyte® S3 software (Essen BioScience). The significance of the differences between the untreated control group (Vehicle) and the groups treated with various compounds was assessed by one-way ANOVA with Dunnett's post-hoc analysis (GraphPad Prism 6.0, Graph-Pad Software, San Diego, CA).

## Supplemental Figures

##
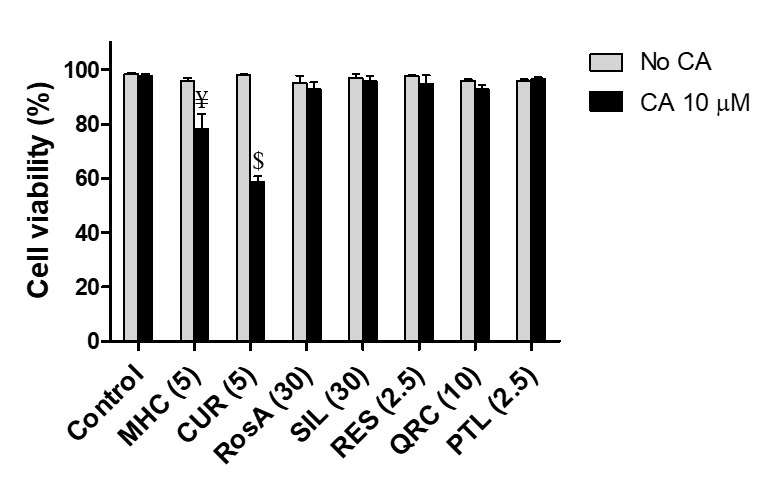


**Supplemental Figure S1**. **Methyl 4-hydroxycinnamate and curcumin, but not other phytochemicals employed in this study are cytotoxic at the chosen concentrations when combined with carnosic acid.** HL60 cells were treated with different phenolic compounds (in μM) : curcumin (CUR), methyl 4-hydroxycinnamate (MHC), rosmarinic acid (RosA), silibinin (SIL), resveratrol (RES), quercetin (QRC), parthenolide (PTL) and/or 10 μM CA, for 72 h. Cell viability was determined by the trypan blue exclusion assay. The data are the means ± SD (n=3). Synergistic effect (AB>A+B): $, p < 0.01; Ψ, p < 0.001, §, p < 0.0001; Student’s t test.


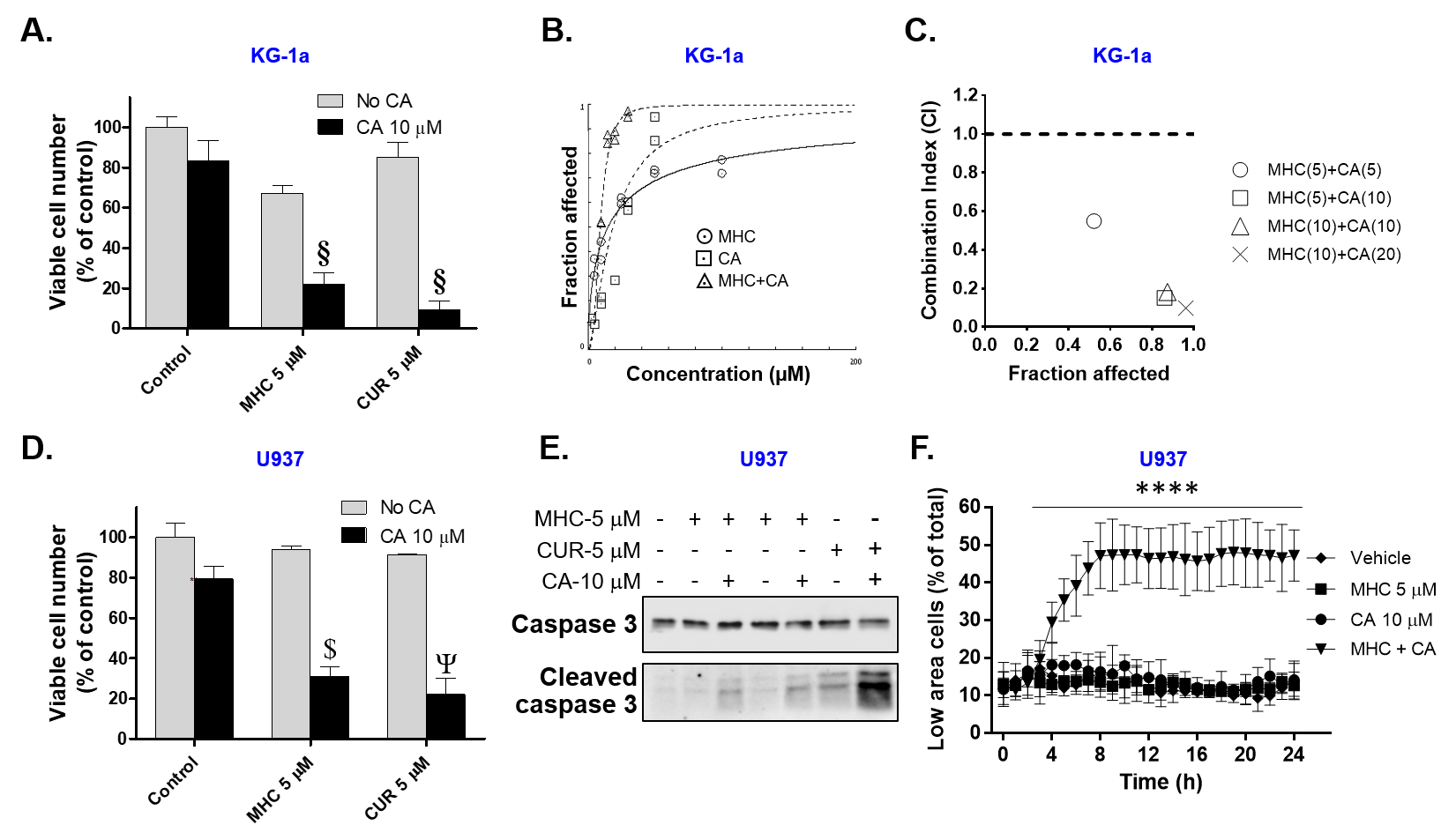


**Supplemental Figure S2. MHC synergizes with CA in inducting anti-leukemic effects in KG-1 and U937 AML cells. (A-D)** Cells were treated with the indicated compounds, for 72 h. **(A)** The number of viable KG-1a cells were counted by the trypan blue exclusion assay. The data are the means ± SD (n=3). **(B)** The extent of reduction in KG-1a viable cell numbers (fraction affected) by MHC and CA, alone and in combination, at the indicated concentrations. **(C)** Combination index (CI) analysis of cytotoxicity for various MHC/CA combinations (μM). The CI values are plotted against the levels of the fraction affected. **(D)** The numbers of viable U937 cells were counted by the trypan blue exclusion assay. The data are the means ± SD (n=3). **(E, F)** U937 cells were treated with the indicated compounds, for 24 h. **(E)** Western blot analysis of caspase-3 cleavage. **(F)** The percentages of low-area cells (<100 μm^2^) were determined during incubation in an IncuCyte® S3 instrument. Synergistic effect (AB>A+B): $, p < 0.01; Ψ, p < 0.001, §, p < 0.0001; Student’s t test. ****, p < 0.0001 vs. Vehicle; **(F)** One-way ANOVA with Dunnet post-hoc analysis.


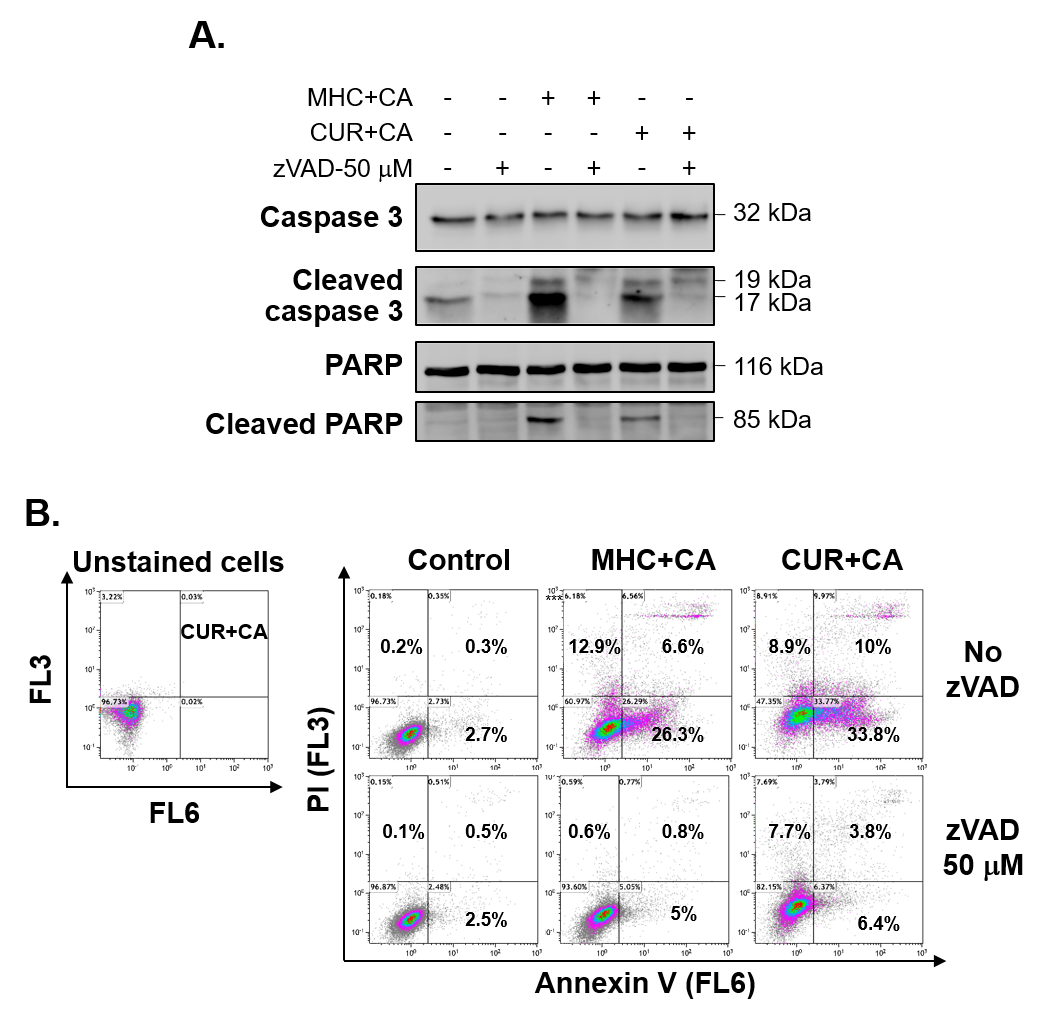


**Supplemental Figure S3. MHC+CA and CUR+CA induce caspase-dependent apoptosis in KG-1a cells.** Cells were treated with the indicated compounds in the presence or absence of zVAD (50 μM), for 8 h. **(A)** Inhibition of caspase-3 and PARP cleavage by zVAD. A representative Western blot analysis (n=3). **(B)** zVAD abolishes the induction of apoptosis (*right panels*). The analysis was performed following the adjustment of the threshold for CUR+CA auto-fluorescence in an unstained cell sample (*left panel*). Representative annexin V/PI binding data (summarized in **Fig. 1B**).

**
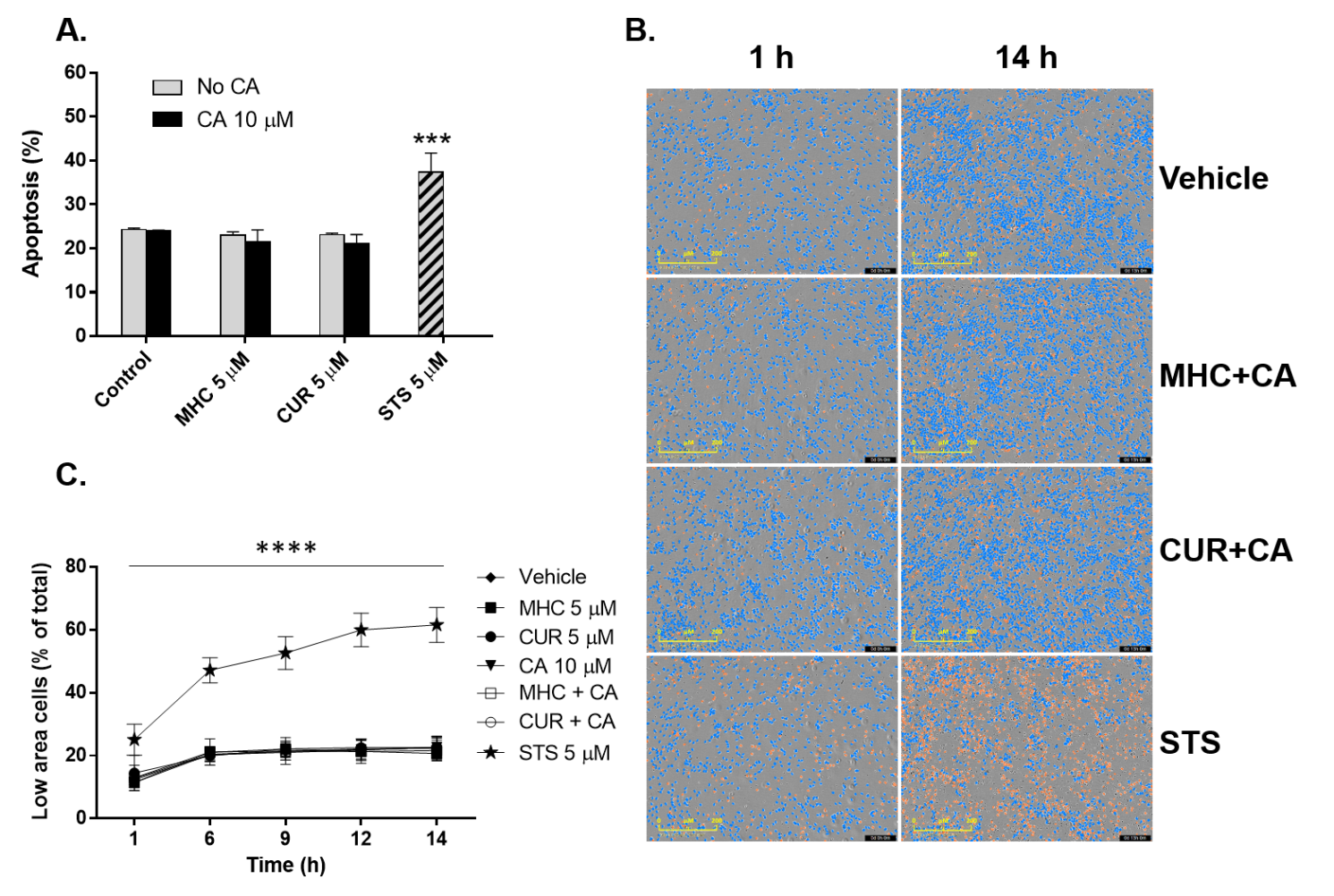
**

**Supplemental Figure S4. MHC+CA and CUR+CA do not kill normal peripheral blood mononuclear cells.** Freshly isolated PBMCs were treated with the indicated compounds for different time periods. The apoptosis inducer staurosporine (STS) was used as the positive control. **(A)** The extent of apoptosis was determined by the annexin V/PI binding assay following 8 h of incubation. **(B)** Representative microphotographs of PBMC cultures following treatment for 1 h and 14 h. Normal-area cells (≥ 50 μm^2^) and low-area cells (< 50 μm^2^) were defined by the IncuCyte® S3 software and are marked in blue and orange, respectively. **(C)** The percentages of low-area cells were determined during incubation in an IncuCyte® S3 instrument. ***, p < 0.001; one-way ANOVA with Tukey multiple comparison post-hoc analysis. **(C)** ****, p < 0.0001 vs. Vehicle; One-way ANOVA with Dunnet post-hoc analysis.


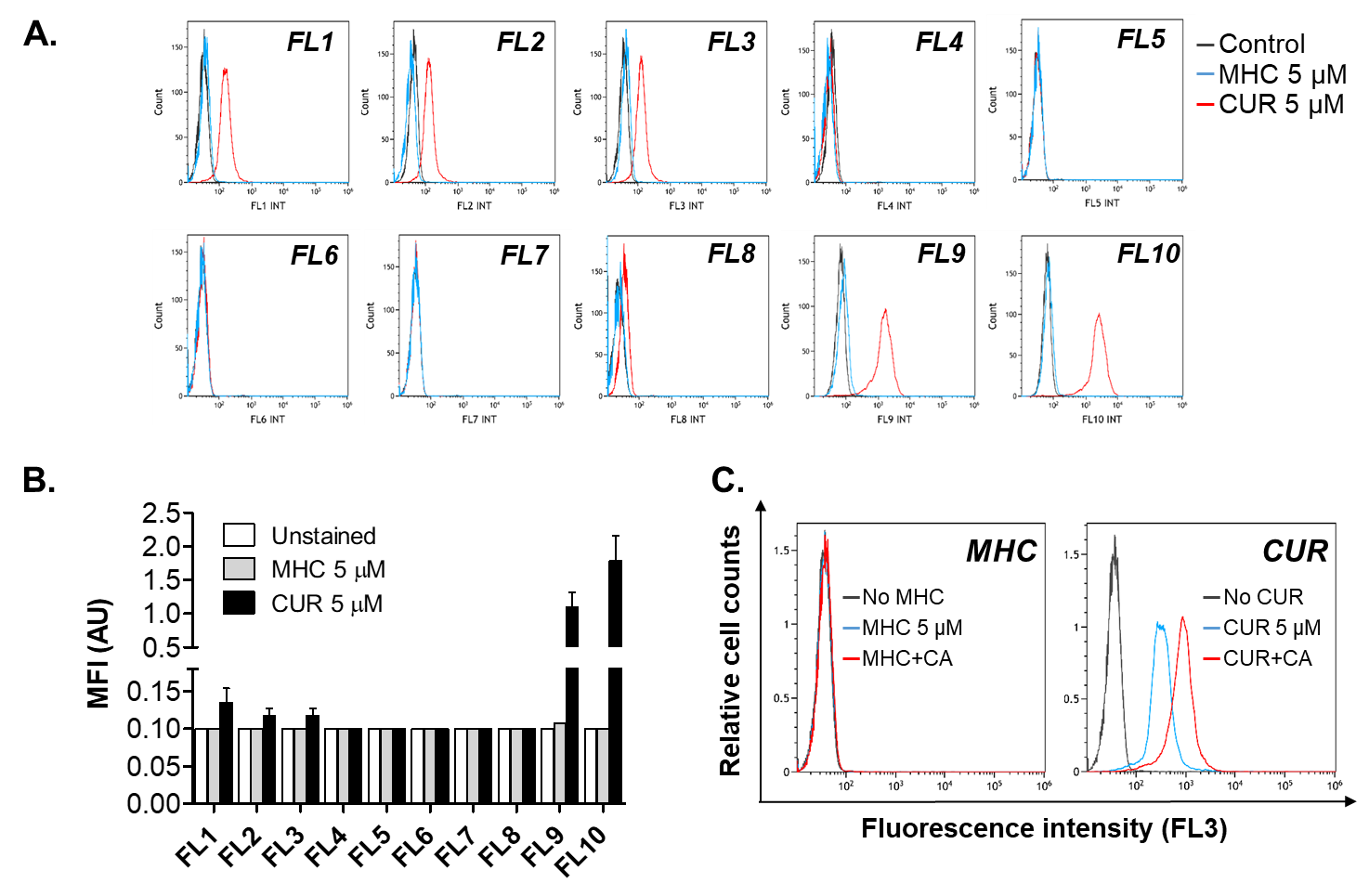


**Supplemental Figure S5. CUR, but not MHC, is fluorescent over a wide range of wavelengths.** HL60 cells were incubated with the indicated compounds, for 24 h, followed by flow cytometric analysis in all the available fluorescence channels (FL1-FL10). **(A)** Typical histogram overlays obtained in a representative experiment. **(B)** Averaged mean fluorescence intensity (MFI) values ± SD (n=4). **(C)** Cells were treated with CUR or MHC in the presence or absence of CA, for 24 h, followed by flow cytometry in FL3 channel. Overlay histograms from a representative experiment in unstained cells are shown. Channel details: (1) Blue laser (488 nm); emission: 505-545 nm (FL1); 560-590 nm (FL2); 605-635 nm (FL3); 680-710 nm (FL4); 755 nm (FL5). (2) Red laser (638 nm); emission: 650-670 nm (FL6); 715-735 nm (FL7); 755 nm (FL8); (3) Violet laser (405 nm), emission: 430-470 nm (FL9); 530-570 nm (FL10). AU – arbitrary units.


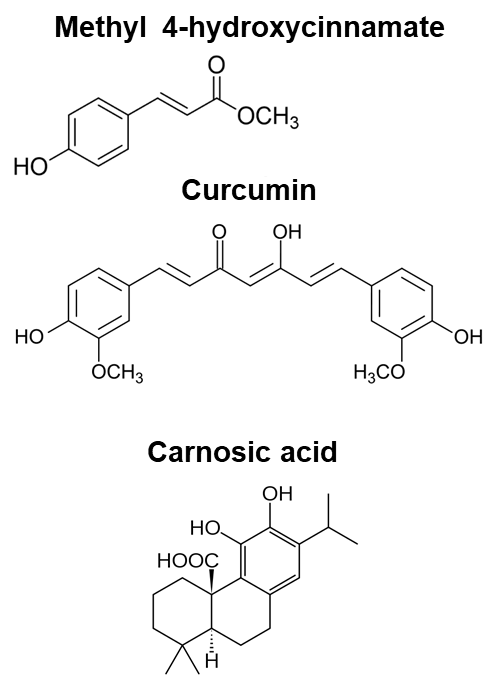


**Supplemental Figure S6. Molecular structures of methyl 4-hydroxycinnamate, curcumin and carnosic acid.**

**Supplementary Videos**

U937 cells were treated with vehicle **(Video 1_v1)** or MHC+CA **(Video 2_v1)**. Following treatments, microphotographs (x200) of U937 cultures were taken once per hour by the IncuCyte® S3 instrument for the period of 24 h and edited into a video file using the IncuCyte® S3 software.

**Supplemental Reference**

Pesakhov, S., Nachliely, M., Barvish, Z., Aqaqe, N., Schwartzman, B., Voronov, E., et al. (2016). Cancer-selective cytotoxic Ca^2+^ overload in acute myeloid leukemia cells and attenuation of disease progression in mice by synergistically acting polyphenols curcumin and carnosic acid. *Oncotarget* 7**,** 31847-31861. doi: 10.18632/oncotarget.7240
